# Trend Impact Analysis (TIA) of community-based futures study for pediatric obesity in Iran

**DOI:** 10.1186/s12887-023-03880-y

**Published:** 2023-02-08

**Authors:** Shahnaz Taghizadeh, Sogol Alesaeidi, Tohid Jafari-Koshki, Seyedeh Masoumeh Valizadeh-Otaghsara, Atousa Poursheikhali, Ayda Zahiri Tousi, Mahdieh Abbasalizad–Farhangi

**Affiliations:** 1 Department of Community Nutrition, Faculty of Nutrition, Tabriz University of Medical Sciences, Tabriz, Iran; 2 Department of Pediatric Medicine, Imam Hossein Hospital, Isfahan University of Medical Sciences, Isfahan, Iran; 3 Department of Statistics and Epidemiology, Tabriz University of Medical Sciences, Tabriz, Iran; 4 Imam Khomeini Hospital, Department of Cancer Institute, Tehran University of Medical Sciences, Tehran, Iran; 5 Health Foresight and Innovation Research Center, Institute for Futures Studies in Health, Kerman University of Medical Sciences, Kerman, Iran; 6 Razavi Cancer Research Center, Razavi Hospital, Imam Reza International University, Mashhad, Iran; 7 Tabriz Health Services Management Research Center, Tabriz University of Medical Sciences, Tabriz, Iran

**Keywords:** Pediatric obesity, Children and adolescents, Trend impact analysis, Futures studies, Iran

## Abstract

**Background:**

Childhood obesity has been regarded as one of the main healthcare challenges in the last century, leading to critical health problems and reduced life expectancy. Many factors can play a role in its development or prevention. Using the Trend Impact Analysis (TIA), this study aimed to conduct a community-based futures study for pediatric obesity in Iran.

**Methods:**

We obtained the prevalence of overweight and obesity from the database of the Ministry of Health and Medical Education. Moreover, we reviewed 21 documents, texts, and comments from three key stakeholders in Iran and prepared a list of key experts, who were stakeholders in the field of obesity prevention of childhood in different organizations. Then, we collected the expert opinions by Delphi method. Data analysis was performed using the Excel and R software.

**Results:**

Fourteen experts participated in the first stage and nine experts in the second stage. We identified two positive drivers, including the prevalence of coronavirus disease 2019 (COVID-19) and the widespread expansion of online educational programs. Meanwhile, we identified five negative drivers as follows: (1) controlling and limiting obesogenic environments in the community, school, and family; (2) running annual compulsory anthropometry programs for students of all educational levels in health centers; (3) integrating nutrition education interventions in the curricula of all educational levels; (4) taxation of unhealthy and fast foods; and (5) preparing safe and appropriate sports environments for children and adolescents (on the streets, schools, parks, and sports clubs). Without considering the drivers, the prevalence of overweight and obesity is predicted to reach 29.10% in 2031. However, it is expected that the negative drivers can increase the prevalence trend from 23.40% in 2018 to 19.57% in 2031, the positive drivers to 32.61%, and the combination of all drivers to 23.07%.

**Conclusion:**

It seems that measures such as the effective communication of policy makers, basic evaluation of the programs and policies related to the prevention of childhood obesity, and localization of the programs of international organizations for the prevention of obesity can greatly control the prevalence of childhood obesity.

**Supplementary Information:**

The online version contains supplementary material available at 10.1186/s12887-023-03880-y.

## Introduction

Childhood obesity is one of the main healthcare challenges in the twenty-first century, leading to serious health problems and reduced life expectancy [1, 2]. Most studies have shown that high-energy diet and sedentary lifestyle are the main causes of the obesity epidemic [3–5]. According to the evidence, interventions that focus solely on dietary change or physical activity cannot be very effective in preventing childhood and adolescent obesity; they can only improve an individual’s diet or increase his/her physical activity. Therefore, changes in diet and physical activity of children and adolescents should be considered along with other factors affecting diet and physical activity [6].

Based on the statistics revealed by the United Nations International Children’s Emergency Fund (UNICEF) in 2016, 124 million children and adolescents are obese, one in five children and adolescents are overweight, and about half of overweight children under the age of five live in Asia [7]. The results of the Childhood and Adolescence Surveillance and Prevention of Adult Non-Communicable Disease (CASPIAN-V) study on 14,274 Iranian students in 2014-2015 showed that 20.6% of children and adolescents aged 7-18 years in 31 provinces of Iran were obese and overweight [8]. Also, a study in 2017 showed that about 14% of Iranian children under 5 years old were at risk of being overweight or suffered from obesity [9].

According to studies, children and adolescents with obesity carry their excess body weight into adulthood [10, 11]; so, preventive interventions in the early stages of life are highly beneficial compared to adolescence and adulthood in terms of costs [12]. The high prevalence of obesity and non-communicable diseases can impose a huge cost and have irreversible effects on the health system and society [13–17]. Due to the increasing prevalence of obesity in this age group, identifying the factors affecting the prevalence of overweight through conducting futures studies can be effective in policymaking related to the childhood obesity prevention [18–20].

Futures studies is a science that uses the analysis of resources, patterns and factors of change or stability to visualize a potential future and plan for them. In fact, future research uses a wide range of methodologies instead of imagining “only one future” to make systematic and wise speculation; it considers “several imagined futures” and analyzes several futures to evaluate the issue [21, 22].

Trend Impact Analysis (TIA), proposed by Theodore J. Gordon in the late 1970s, is one of the methods used in futures studies. TIA is suitable for combining quantitative and qualitative methods. It is a forecasting method that permits extrapolations of historical trends to be modified in view of expectations about future events. TIA uses quantitative and numerical methods for its predictions to improve the initial quantitative prediction by using possible future events (qualitative) from the experts’ point of view (for example, using the Delphi method). The events can have a wide span to include technological, political, social, economic, and value-oriented changes [23].

Accordingly, considering the high prevalence of obesity and overweight in children and adolescents in Iran, futures studies using TIA method and identifying the factors affecting the prevalence of obesity and its impact on this trend can help Iranian policymakers to make appropriate decisions.

## Materials and methods

**Fig. 1:**
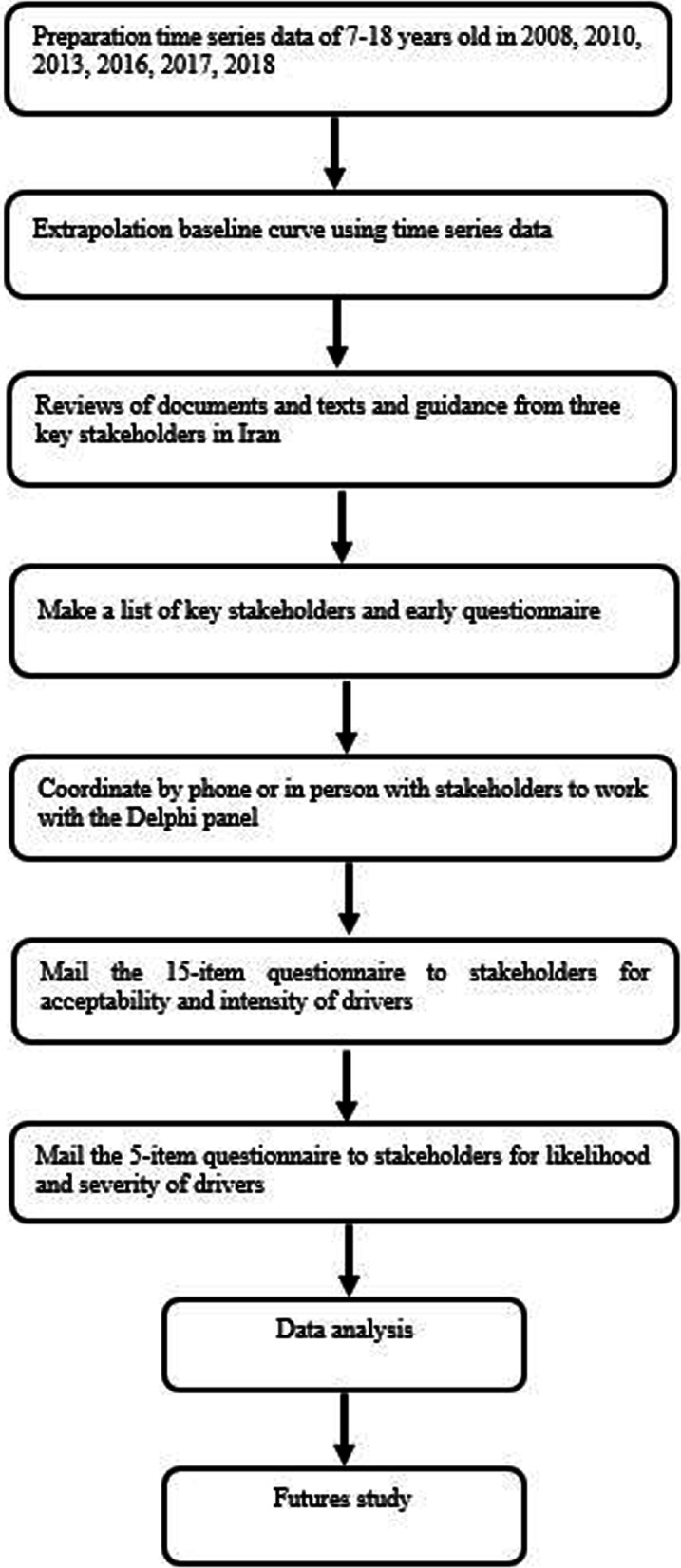
Flow chart of study

This qualitative study was part of a comprehensive study entitled “Futures Study and Policy Analysis of the Prevention of Obesity in Children and Adolescents in Iran and Providing Policy Options”. We reviewed the documents, texts, and comments from three key stakeholders in Iran. Then, several key experts working in the field of childhood obesity prevention were selected purposefully. TIA was performed using Delphi method to collect specialized information of experts and time series data on the prevalence of overweight and obesity in children and adolescents (Fig. 1). TIA consists of two steps. In the first step, a baseline forecast is generated using a suitable statistical model based on historical data. In the second step, a set of future events and their impacts are identified through using prior knowledge elicited from experts or collecting information by qualitative forecasting approaches like Delphi Survey. This is followed by applying Monte Carlo simulation in the TIA algorithm, which combines the impact and event probability judgments with the outcomes of the baseline scenario to generate a possible future scenario. TIA approach has previously been applied in the field of health for predicting future fruit consumption in the Netherlands [24].

### Collection of time series data

Due to the lack of national data on the prevalence of obesity and overweight for the age group of less than 6 years, this study was conducted based on time series data of CASPIAN studies conducted on the age groups of 7-18 years in 2008, 2010, and 2013. Also, the integrated health system (IHS) (known as “SIB” system) of the Ministry of Health and Medical Education was used to access the data of the health system transformation plan in 2016, 2017, and 2018. Due to the coronavirus disease 2019 (COVID-19) pandemic, it was not possible to fully assess overweight and obesity in 2019; hence, the data related to this year were not evaluated (Table 1).

**Table 1:** Prevalence of overweight and obesity in children and adolescents

Year	Prevalence of obesity and overweight ^**a**^(%)	Sample size	Age range (years)
2008	17.6	5088	10-18
2010	21.2	13,487	7-18
2013	20.6	14,274	7-18
2016	21.7	344,682	7-18
2017	23.1	280,260	7-18
2018	23.4	282,165	7-18

^a^According to World Health Organization criteria

### Identifying the drivers of the prevalence of overweight and obesity in children and adolescents

This step was performed by Delphi method. The proposed drivers had to be community-based, acceptable, have a strong impact, be retrospectively predictable, and have no effects on the prevalence of obesity and overweight heretofore in Iran. In this regard, Theodore J. Gordon mentioned that unprecedented events should be selected [23].

The probability of happening the drivers is not certain, and according to experts, these drivers are likely to happen in the coming years. It is possible that they did not happen; so, these possibilities are specified in the questions based on the opinions of experts.

The retrospective predictability of the events was obtained from the documents, texts, and the results of a review study on obesity prevention policies in children and adolescents [25]. Then, we added several other drivers by reviewing a study on identifying the barriers and facilitators of obesity prevention policies in children and adolescents [26]; these cases had been identified as barriers and facilitators in other parts of the world, though they did not have an effect on obesity prevention policies for children and adolescents in Iran [27]. Each confounding factor was also considered to have a positive or negative impact.

The initial list, as a 15-item questionnaire, was sent to the members of the Delphi panel by email for acceptability and intensity supplementary file (B). The online Delphi method can greatly reduce the biases resulting from face-to-face meetings [28]. At this stage, participants were asked to express the probability of occurrence of each of the above events in percentage (from zero to 100) in each of the mentioned years (from 2021 to 2031). Also, they were requested to score the severity of the effect of the events on the prevalence of overweight and obesity in children and adolescents based on a Likert scale from 0 to 10 (the lowest intensity = 0 vs. the highest intensity = 10). According to the results of the first stage of Delphi, if an event had an average intensity of less than 3 or, according to the experts, had already affected the prevalence of obesity among children and adolescents, it did not enter the second stage. Considering the Delphi studies using a Likert scale, which enters the second phase, the questions with less than 25% agreement did not enter the second phase of Delphi. Moreover, since the Likert scale in questionnaire was not based on percentage, we could not remove number 2.5 (which is equivalent to 25%). Hence, according to the opinion of the study team, the cut-off point was set at 3, and the questions that scored less than 3 did not enter the second phase [29].

Other events, as potential drivers causing the future trend to deviate from the unforeseen state, entered the second stage of Delphi questionnaire after approval by the research team and three experts. It should be noted that the experts were informed about the aim of study and how to answer the questionnaires in both stages by phone, during the preliminary interview with the researcher, and guidelines in the email. In the questionnaire, experts were asked to express the likelihood of driver occurring as a new but effective event, as well as the severity of its effect (‘+’ sign for increased prevalence of overweight and obesity [positively influencing driver] and ‘-’ sign for reduced prevalence of overweight and obesity [negatively influencing driver]) from 2021 to 2031 for each year separately. In this study, we employed the Standards for Reporting Qualitative Research (SRQR) to describe the design and findings [30] (supplementary file A).

### Data analysis

First, we assessed the data to find the best predictive baseline model of future prevalence. After fitting various models, linear regression showed the best fit with *R*^2^ = 0.80 and adjusted *R*^2^ = 0.75. Then, this baseline model was combined with data extracted from expert opinions to generate predictions of future overweight and obesity prevalence. For this, according to Theodore Gordon [23] and the Millennium Project (https://www.millennium-project.org), Monte Carlo simulations with random values of 10,000 repetitions were used for all variables [23], and a probability distribution was defined for all of them. Here, for each event of interest we follow the following algorithm. Stating from the first year in prediction course, we generated a random value from Uniform distribution, r ~ Uniform (0, 1) for each year. If the randomly generated value of r for each year exceeded the average occurrence probability of corresponding event in the corresponding year, then the impact of that event was algebraically added to the total impact in that year, as well as to following years until the last year of prediction. Otherwise, algorithm moved to the next year and the loop was repeated until the last year. In this way, the impact of each event was calculated over years of prediction. This constituted one repetition and algorithm was repeated 10,000 times for each event. The whole algorithm was repeated over all events and impacts from different events were added for each year and overall impact of all events was stored in a matrix of 10,000 × years. In the final step, median, 2.5th, and 97.5th percentiles were calculated for each year (column) and added to the baseline prediction to obtain simulated 95% confidence interval of prevalence prediction over the upcoming years. All simulations were conducted in open source and free statistical software R (https://cran.r-project.org).

## Results

### Time series data

**Fig. 2:**
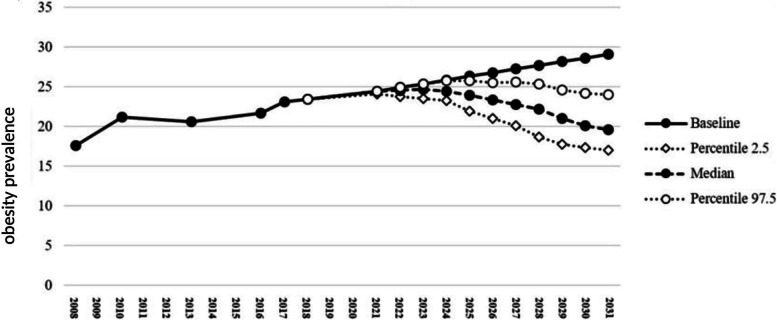
The effects of drivers negatively affecting the trend of overweight and obesity in children and adolescents in the next 10 years

**Fig. 3:**
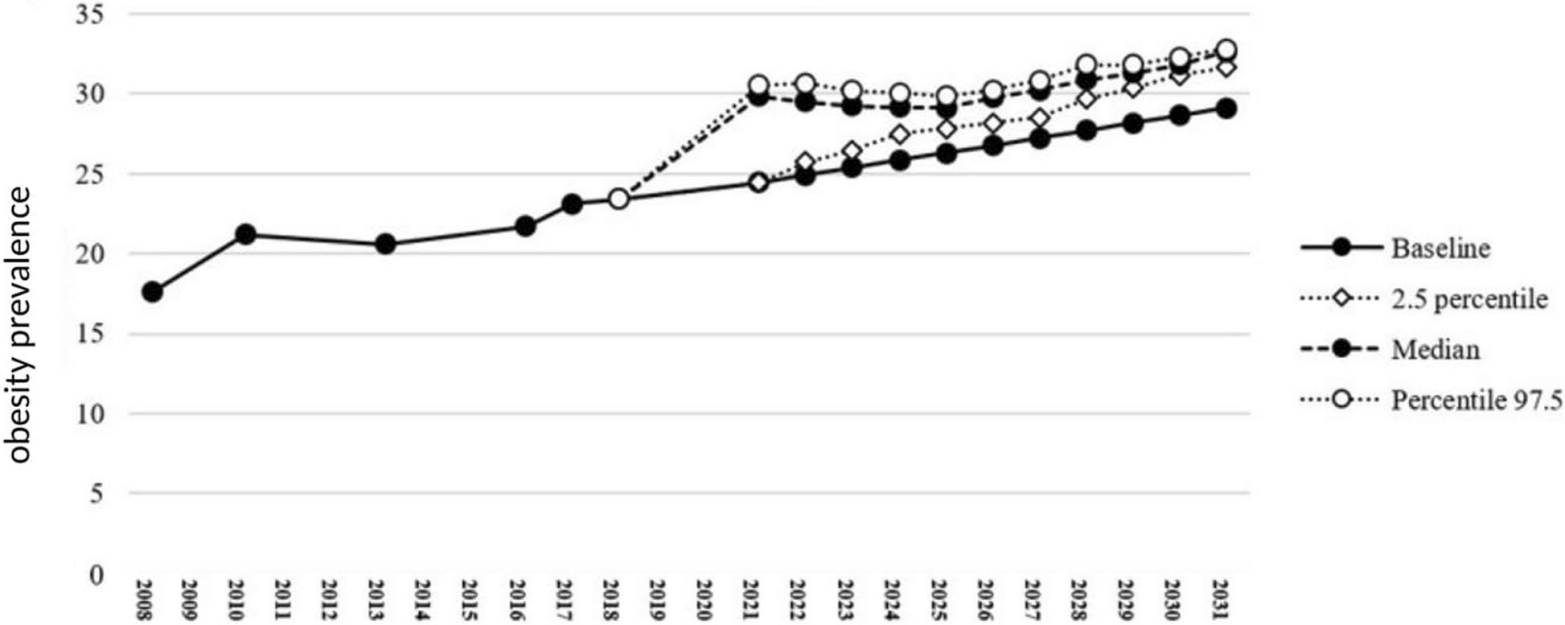
The effects of drivers positively affecting the trend of overweight and obesity in children and adolescents in the next 10 years

**Fig. 4:**
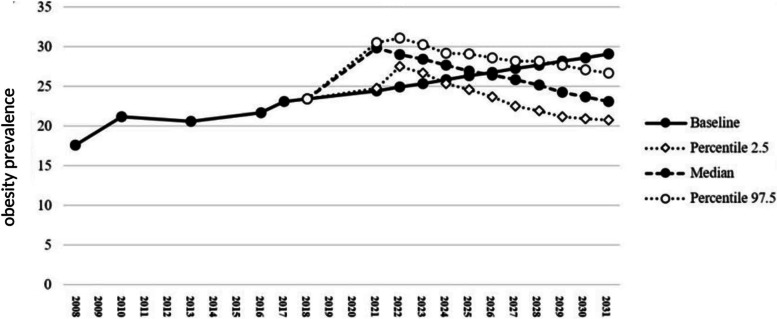
The effects of drivers affecting the trend of overweight and obesity in children and adolescents in the next 10 years in both negative and positive manners

Table 1 shows the data related to the prevalence of overweight and obesity from CASPIAN studies, as well as information from the SIB system. Using the above information, a baseline curve was plotted, which showed that without considering the drivers, the prevalence of overweight and obesity is predicted to reach 29.10% in 2031 (which is shown as the baseline in each of the three Figs. 2, 3 and 4).

### Identifying the drivers affecting the prevalence of overweight and obesity in children and adolescents

The results of the first stage of Delphi led to the identification of seven drivers of overweight and obesity in children and adolescents in the 10-year horizon in Iran; two drivers were identified as positive drivers and five drivers as negative drivers. The two positive drivers, which may increase the risk of obesity among children, included COVID-19 disease (continuation of the pandemic) and the widespread expansion of online educational programs (cultural, artistic, and related to the lesson). On the other hand, negative drivers included: (1) controlling and limiting obesogenic environments in the community, school, and family; (2) running annual compulsory anthropometry programs for students of all educational levels in health centers; (3) integrating nutrition education interventions in the curricula of all educational levels; (4) taxation of unhealthy and fast foods; and (5) preparing safe and appropriate sports environments for children and adolescents (on the streets, schools, parks, and sports clubs).

### The effect of drivers on the prevalence of overweight and obesity in children and adolescents

The mentioned drivers entered stage 2 of Delphi panel to be scored, so that their probability and intensity are ranked in percentage. Three different types of curve (positive impact, negative impact, and a combination of both drivers) show the significant impact of these drivers over the next 10 years, if they happen. The present study showed that positive and negative drivers changed the estimated values of prevalence trend. Figure 2 shows the effect of five negative drivers on the prevalence of obesity among children and adolescents. It is estimated that these drivers may change the prevalence of overweight and obesity from 23.40% in 2018 to 23.33% in 2026 with a confidence interval of 21.01 to 25.53. Also, at the end of the estimation period in 2031, it might reach 19.57% with confidence interval of 17.02 to 24.05. A different curve was obtained for the two positive drivers. The curve of positive drivers (Fig. 3) showed a potential significant increase in the prevalence of overweight and obesity as a result of these two drivers. So, it is expected that the mentioned drivers can change the prevalence from 23.4% in 2018 to 29.77% with a confidence interval of 28.16 to 30.22 in 2026 and 32.61% with a confidence interval of 31.64 to 32.79 in 2031. It should be noted that both positive and negative drivers exist in the real world and simultaneously affect the trend. Therefore, by combining these drivers, Fig. 4 is obtained, which indicates that these drivers can decrease the prevalence of overweight and obesity to 26.41% with a confidence interval of 23.67 to 28.59% in 2026 and to 23.07% with a confidence interval of 20.80 to 26.65 in 2031. In the first stage of the panel, 14 experts participated, of whom nine were willing to participate in the second stage (participation rate: 64.2%). Table 2 shows the details of the participants in the first and second stages of the panel.

**Table 2:** Organizational characteristics of the participant

Participating organization	Subset of participating organization	1^st^ Stage of Delphi (*n* = 21)	2^nd^ Stage of Delphi (*n* = 15)
MoHME^a^	Health Deputy of MoHME	1	1
	Professor of university of medical sciences	7^b^	6^c^
	Department of Community Nutrition Improvement of Provincial Health Center	2	1
	Department of Non-Communicable Diseases of the Provincial Health Center	1	–
	Physician of Health Center	1	
Ministry of Education	Executive Manager of Ministry of Education	1	1
	Provincial Executive Manager	1	–

^a^Ministry of Health and Medical Education

^b^2 nutritionists, 1 pediatrician, 1 health policy specialist,1 health education specialist, 1 Food industry specialist, 1 food and nutrition policy specialist

^c^2 nutritionists, 1 health policy specialist, 1 pediatrician, 1 food and nutrition policy specialist, 1 health education specialist

## Discussion

Given that most of the factors that seem to increase the prevalence of overweight and obesity in children and adolescents have been effective on the trend of overweight and obesity, only two drivers were selected as new drivers by the experts. One of the most important positive drivers was COVID-19 (continuation of the pandemic), the effect of which on the prevalence of childhood overweight and obesity has not been documented yet. This case has been considered as an effective event and the extent of its effect has been considered for subsequent years. Studies show that the outbreak of COVID-19 led to socio-economic changes at the community level [31], increased intake of high-energy foods, and reduced physical activity in children and adolescents, all of which predisposed them to obesity [32]. Therefore, if we consider the effects of COVID-19 outbreak, they may have adverse effects on public health. Although COVID-19 has been partially controlled, studies show that a rebound effect in transmission could occur after several years and if it spreads again, the subsequent health problems will increase [33]. The other potential positive driver is the widespread expansion of online educational programs (cultural, artistic, and related to the lesson), and consequently the reluctance to attend physical activity and sports programs due to the fear of infectious diseases. As a result, children, adolescents, and families might get used to sedentary lifestyle in the coming years, which may increase obesity. However, Neve et al. showed that appropriate use of online programs such as web-based weight loss and weight loss maintenance interventions have the potential to achieve results similar to other lifestyle treatment options [34].

Some other models have been used to predict the trend of obesity in the future, which have examined only one of the dimensions affecting the trend of obesity. For example, Chen et al. using the system dynamics modeling, showed that improving people’s income from lower to middle-income group would help control the rising prevalence of obesity in the future [35]. One of the most important differences between the TIA and most of the other models is that in those models analysis is merely based on past trends and historical data, or only experts’ opinions and qualitative data.

The first negative driver according to the opinion of stakeholders, which may affect the trend of children’s obesity in the future, is controlling and limiting obesogenic environments in the community, school, and family. Lack of safe distance from unhealthy food shops near schools has been identified as one of the important threats of the childhood obesity prevention policy. Moreover, the lack of a suitable and safe place for physical activity and high advertising of unhealthy food in the mass media are the most important factors contributing to overweight and obesity in Iran. Studies show that more than 60% of television commercials in Iran are related to food [36], most of which include unhealthy foods that strongly affect the food choices of children and adolescent [37]. A review study by Jia et al. showed that obesogenic environments can increase the prevalence of obesity by restricting the physical activity of children and adolescents [38]. Schroeder et al. stated that consequences of obesogenic environments, which can be affected by economic, social, and cultural conditions, increase the consumption of high-energy foods and lead to inactivity in children and adolescents, and consequently increase the prevalence of obesity and prevent optimal implementation of childhood obesity prevention policies [39]. Therefore, individual behavioral changes to prevent overweight and obesity require staying away from obesogenic environments.

The second negative driver identified in this study was running annual compulsory anthropometry programs for students of all educational levels in health centers. The compulsory anthropometry measurement in Iran is performed for four times (in the first, fourth, seventh, and tenth levels) in health centers. In addition, most families are unaware of the importance of weighing their children [27]. Thus, the lack of screening weight status for several years may increase the risk of obesity. Similar to our study, Cyril et al. showed that the lack of anthropometric measurement of all students in schools is one of the problems of obesity prevention, which can increase the prevalence of obesity [40]. Due to the insufficient healthcare staff in schools, it seems that providing primary healthcare at the beginning of the school year for all educational levels by the health system can be effective in the early detection of overweight and its transformation into obesity in this age group [41].

Another effective factor in the future of children’s obesity was related to integration of nutrition education interventions in the curricula of all educational levels. There are few nutrition lessons in Iranian schools, and the only grade including a separate unit on health and nutrition education is the twelfth grade. Since students take the university entrance exam at this level and this course is not part of the entrance exam, it does not have much effect on students’ knowledge and performance. Hayes et al. showed that nutrition and physical activity training programs, if implemented in the form of educational and training curricula, can overcome the obstacle of interfering with school hours and have a greater effect on the prevention of obesity [42].

The fourth driver in our study was taxation of unhealthy foods and fast foods. Value-added tax (VAT) has been imposed only on fast food chain restaurants since early 2015 in Iran, and other fast food restaurants are not allowed to receive VAT from the customers [43]. The policy implications of taxing unhealthy foods have been shown in many studies. Cobiac et al. and Powell et al. showed that taxing unhealthy foods and providing them at higher prices could reduce the demand from most children and adolescents and it played a major role in preventing childhood and adolescent obesity [44, 45]. Using a futures study in 2012, Kristensen et al. found that of the 26 policies surveyed to prevent childhood and adolescent obesity, taxing sugary drinks could reduce the prevalence of obesity more than any other policies for the next 20 years in the age group of 13-18 years [46].

Finally, we identified preparing safe and appropriate sports environments for children and adolescents (on the streets, schools, parks, and sports clubs) as the fifth negative driver. Most studies have highlighted that having safe and appropriate sports environments can increase physical activity, improve mental health, and reduce weight self-stigma in children and adolescents [17, 42, 47, 48]. It is expected that in adopting policies such as the Shafa plan, the active cities in Iran that focus more on promoting physical activity for adults pay more attention to children and adolescents, the use of sports centers and gyms, and the effective implementation of physical education course in schools.

In general, the prevalence of overweight and obesity after 10 years will probably be a steady trend with the effect of positive and negative drivers studied in the present study. Although the status of overweight and obesity in children has not been discussed much in national documents, in the National Document for the Prevention of Non-Communicable Diseases, one of the expected goals is to keep the prevalence of obesity in adults constant until 2025. The present study predicts the achievement of this perspective in the age group of children and adolescents in the next 10 years. In the present study, where future uncertainties must be considered, there is a need for a quantitative forecasting method in which a time series is modified to consider future uncertainties. Among the existing forecasting methods, these requirements can be met by TIA and CIA (cross impact analysis). TIA identifies a set of important future events that can deviate from the extrapolation of historical data, judges their probabilities and impacts, and thus forecasts a range of future values rather than a single point [49]. CIA estimates the potential interactions among future events and adjusts the probabilities of occurrence [50]. When experts have little confidence in quantifying such interactions, TIA is better than CIA [51].

In a Canadian study, Twells et al. examined obesity in adults using trend analysis and found that between the years 1985 and 2011, the overall prevalence of obesity ranged from 6.1 to 18.3%; and the prevalence of Grade 1 obesity increased from 5.1 to 13.1%, Grade 2 obesity from 0.8 to 3.6%, and Grade 3 obesity from 0.3 to 1.6%. Therefore, using trend analysis, they predicted that the prevalence of Grade 1, 2, and 3 obesity would reach 14.8, 4.4, and 2% by 2019, respectively; however, they did not examine the factors that could possibly change this trend [52]. Andersen et al. showed that the child’s primary care provider in the identification of overweight is the most important factor in preventing childhood obesity. Moreover, obesity-related knowledge gaps and affordability of healthy foods and activities were the largest barriers to helping Latino children maintain healthy weights [53]. The disagreement between our results and those reported by Andersen et al. might be attributed to the fact that in their study obesity of children under 5 years old was considered and only qualitative Delphi method was used and quantitative data was not used in the prediction model. One of the most important strengths of the TIA method is that the analyst states what events will cause change in the future. In fact, in the TIA method, quantities are added to a scenario at the same time as qualitative results. Because TIA provides a range rather than a single point prediction, uncertainty can be explicitly considered in decision analyses. Therefore, TIA fits well with risk analysis techniques.

One prospective study in 2012 examined the effect of after-school physical activity program, which is one of the most important childhood and adolescent obesity prevention policies in the United States, on weight of children and adolescents. It was estimated that by 2035, the plan would lead to the greatest decline of obesity among children aged 6 to 12 (with a 1.8% reduction in obesity) [46]. Another study used the trend analysis model based on expert knowledge and historical data on fruit consumption; the researchers applied TIA for four European countries and identified ‘achieving the desired level of health’, ‘achieving the desired comfort and facilities’, and ‘using initiative and diversity in production’ as three drivers with positive effect on fruit consumption. They also expected that fruit consumption would increase by 2.12 kg per year from 2007 to 2025 due to the influence of the mentioned drivers [24].

## Limitations

This study had some limitations. First, since TIA is relatively less known and to some extent a difficult method, its application requires a lot of time and patience on the part of the researcher. Experts seem to have difficulty in obtaining the types of questions, and it is essential that experts, as knowledgeable individuals, participate in this method of analysis to be fully aware of the policies analyzed in the past and future. Also, coordination with such experts is a time-consuming issue. Second, this study coincided with the COVID-19 pandemic, which prolonged Delphi phase. Third, some organizations refused to cooperate even in the first phase of the study and some of the stakeholders in the first stage refused to participate in the second stage. Fourth, the points the stakeholders gave to the drivers based on the ranking were based on judgments that may be affected in a period such as the period of economic sanctions against Iran.

## Conclusion

The identified effective drivers and their impact on the prevalence of overweight and obesity in children and adolescents can be used by policy makers, managers, and decision makers in the prevention of childhood obesity prevention policies in Iran.

## Supplementary Information

**Additional file 1: Supplementary File A.** COREQ (COnsolidated criteria for REporting Qualitative research) Checklist.

**Additional file 2: Supplementary File B.** The initial list of 15 drivers which experts selected from for context.

## Data Availability

All of the data are available with reasonable request from the corresponding author.
