# Supplementary material for: Trend Impact Analysis (TIA) of community-based futures study for pediatric obesity in Iran
Source: BMC Pediatr. 2023 Feb 8;23:66. doi: 10.1186/s12887-023-03880-y (PMC9905010; doi:10.1186/s12887-023-03880-y)
Supplement: Supplementary file 2 — Additional file 2: Supplementary File B. The initial list of 15 drivers which experts selected from for context. [file 12887_2023_3880_MOESM2_ESM.pdf]

supplementary file (B). The initial list of 15 drivers which experts selected from for context

1. Increasing the awareness and attitude of stakeholders (children, parents, program executives and policy makers) regarding obesity and its prevention
2. Sufficient participation and cooperation of stakeholders (executives, policy makers, children and parents)
3. Sufficient manpower Providing in the policy implementation level
4. Proper monitoring and control of the announced policies for implementation
5. Providing equipment and facilities (such as sufficient financial resources to provide free food or sufficient sports and educational facilities)
6. Mandatory annual weight control for all school students
7. Controlling and limiting obesogenic environments in the community, school, and family environment
8. Creating safe and suitable sports environments for children and adolescents (in streets, parks and sports clubs)
9. Integration of intervention programs with the school curricula
10. Tax Imposing on, fast foods, ready-to-eat foods and sugar-sweetened beverages (increasing them price)
11. Implementation of intervention programs during children's non-school hours (for example, nutrition education or provision of sports programs in the afternoons)
12. Regular and daily distribution of milk in school
13. Low salaries of executive beneficiaries of childhood obesity prevention programs
14. Increasing academic pressure in children and adolescents
15. Widespread advertising encouraging the consumption of ready-to-eat foods (foods with low nutritional value and high energy) for children and adolescents
